# Global financing retrenchment threatens the recovery in TB control

**DOI:** 10.5588/ijtldopen.26.0371

**Published:** 2026-10-12

**Authors:** F. Chen, Y. Zhang, L. Chen

**Affiliations:** Department of Child Healthcare, Wenzhou People’s Hospital, The Third Clinical Institute Affiliated to Wenzhou Medical University, Wenzhou, Zhejiang, China.

**Keywords:** tuberculosis, health expenditures, foreign aid, resource allocation, public health preparedness, drug resistance

Dear Editor,

We read with interest the recent article by Yassin et al., which highlights evidence-based, cost-effective strategies to optimise TB programmes under funding constraints.^1^ The authors argue that approaches such as service integration, technological innovation, community engagement, and domestic resource mobilisation can improve programme efficiency and help sustain TB services despite the widening funding gaps. Their analysis highlights that optimisation is increasingly viewed as a means of preserving recent gains in TB control. However, optimisation cannot substitute for sustained financing. After the profound disruptions caused by the COVID-19 pandemic, global TB control has been recovering, with modest declines in both incidence and mortality.^2^ As progress resumes, the contraction in international financing (including reductions in bilateral aid, restructuring of global health programmes, and constrained replenishment of mechanisms such as the Global Fund) suggests an inflection point.^3^ The growing mismatch between TB control needs and available resources is shown in the Figure.

**Figure.**
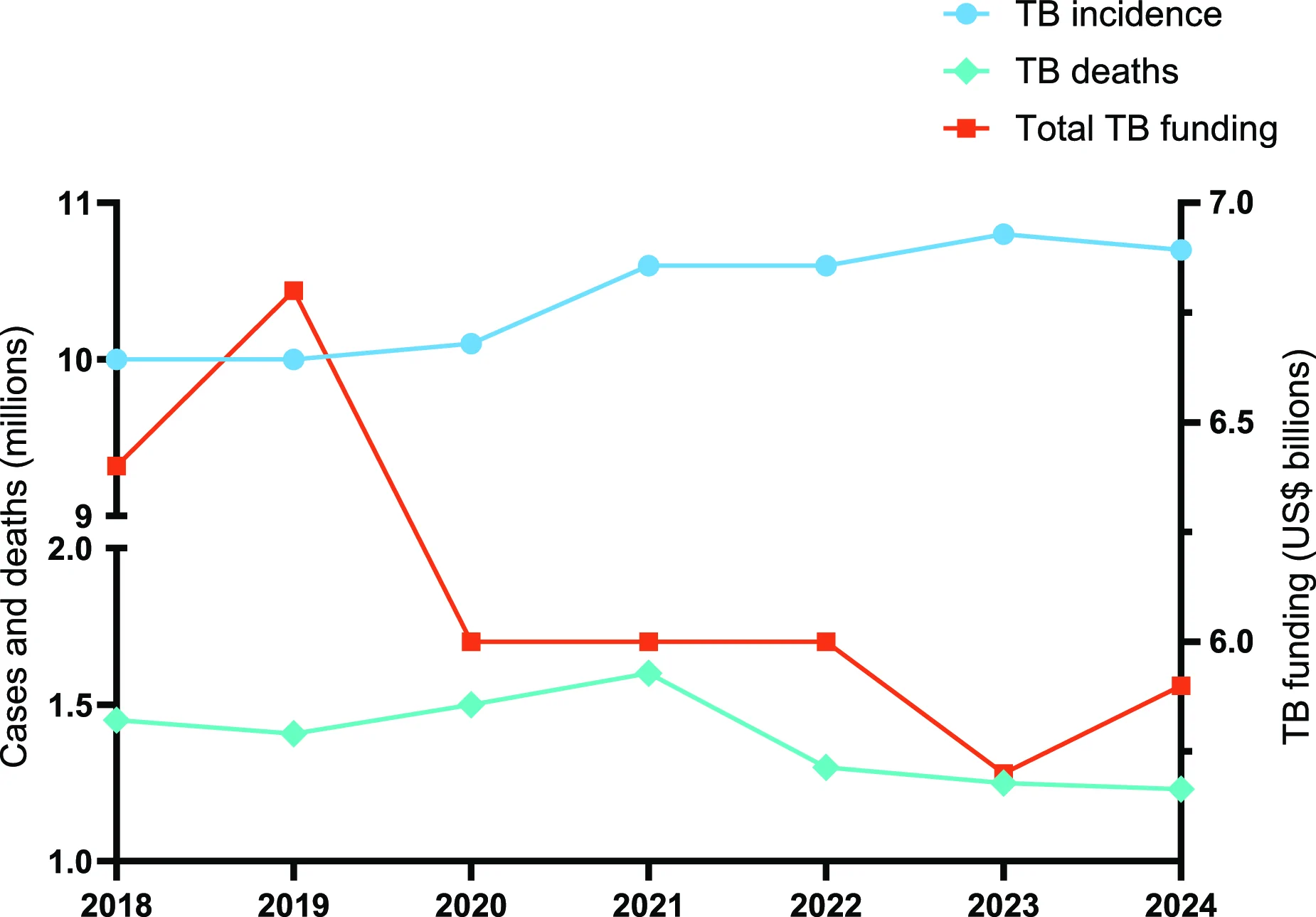
Trends in global TB burden and total TB funding, 2018–2024. Annual estimates of TB incidence and TB deaths were obtained from the WHO Global Tuberculosis Report 2025. Total TB funding data were obtained from the WHO Global Tuberculosis Report 2025 and represent the total funding available for TB prevention, diagnosis, treatment, and care, including both domestic and international sources. Data were extracted directly from published reports and plotted without additional transformation, normalisation, or statistical modelling.

Despite progress, TB remains a leading infectious cause of death, with an estimated 10.7 million incident cases and 1.23 million deaths in 2024, including 150,000 among people living with HIV.^2^ Sustained control depends on investment in diagnosis, treatment, contact tracing, and prevention, which in many high-burden settings remain heavily reliant on external funding.^2^ Disruptions delay diagnosis, sustain transmission, and accelerate drug resistance. The COVID-19 pandemic showed how even short-term interruptions can reverse years of progress.^4^ Early evidence from high-burden settings suggests that reduced community screening and outreach have led to fewer diagnoses at earlier disease stages.^5^ Similar disruptions may now arise not from epidemiological shocks but from financial constraints.^3^ Global TB funding has declined substantially and remains far below internationally endorsed targets.^3,6^ These reductions are not routine fiscal adjustments; they directly reduce the operational capacity of TB programmes.^7^ In several high-burden countries, funding shortfalls have already weakened community services and programme capacity.^7^ These developments reflect broader shifts in global health investment priorities.^3,6,8^ Although TB incidence has begun to decline after pandemic-related increases, progress remains insufficient to meet global targets.^2^ Unlike acute outbreaks that prompt rapid mobilisation, TB control requires sustained system capacity. The consequences of disruption may accumulate over time as increased transmission, mortality, and drug resistance. In this context, funding contraction is a structural threat to fragile gains.

A key policy challenge is how TB is positioned within evolving global health agendas. Recent investment in pandemic preparedness and emerging threats has often been accompanied by shifting financing priorities.^8^ Yet, framing preparedness as separate from ongoing disease control is a false dichotomy. TB is itself a persistent global health emergency. Weakening TB programmes risks undermining diagnostic systems, surveillance capacity, and community-based care that are essential for preparedness. Integrating TB control into preparedness frameworks would better reflect this continuity. Addressing these risks requires strategic policy adjustment and reframing TB as a protected component of global health security. First, major funding mechanisms should establish minimum protected financing thresholds for TB. Second, high-burden countries should be supported through co-financing and technical assistance to strengthen domestic investment.^5^ Third, TB service indicators should be incorporated into preparedness metrics, ensuring that case detection, treatment coverage, and drug supply continuity are preserved during fiscal constraint.

In conclusion, the recent recovery in TB control shows that progress is achievable when political commitment aligns with sustained financing. Allowing this progress to erode through funding contractions would represent a failure of equity and strategic foresight. Sustained investment in TB is not merely a response to a longstanding disease burden; it is a test of whether global health systems can maintain commitment to enduring threats while preparing for emerging ones.
